# Equine Encephalosis Virus

**DOI:** 10.3390/ani12030337

**Published:** 2022-01-29

**Authors:** Sharon Tirosh-Levy, Amir Steinman

**Affiliations:** 1 Koret School of Veterinary Medicine, The Robert H. Smith Faculty of Agriculture, Food and Environment, The Hebrew University of Jerusalem, Rehovot 7610001, Israel; amirst@savion.huji.ac.il; 2 Division of Parasitology, Kimron Veterinary Institute, Beit Dagan 50200, Israel

**Keywords:** equine encephalosis virus, EEV, horse, epidemiology, clinical disease, control, *Culicoides*

## Abstract

**Simple Summary:**

Equine encephalosis (EE) is a febrile disease of horses caused by EE virus (EEV) and transmitted by *Culicoides* midges. This virus was first isolated from a horse in South Africa in 1967 and until 2008 was believed to be restricted to southern Africa. In 2008–2009, isolation of EEV in an outbreak reported from Israel demonstrated the emergence of this pathogen into new niches. Indeed, further testing revealed that EEV had already spread outside of South Africa since 2001. Although EEV normally does not cause severe clinical disease, it should be considered important since it may indicate the possible spread of other related, much more pathogenic viruses, such as African horse sickness virus (AHSV). The spread of EEV from South Africa to central Africa, the Middle East, and India is an example of the possible emergence of new pathogens in new niches and should be a reminder not to limit the differential diagnoses list when facing a possible outbreak or a cluster of undiagnosed clinical cases. This review summarizes current knowledge regarding EEV structure, pathogenesis, clinical significance, and epidemiology.

**Abstract:**

Equine encephalosis (EE) is an arthropod-borne, noncontagious, febrile disease of horses. It is caused by EE virus (EEV), an *Orbivirus* of the Reoviridae family transmitted by *Culicoides*. Within the EEV serogroup, seven serotypes (EEV-1–7) have been identified to date. This virus was first isolated from a horse in South Africa in 1967 and until 2008 was believed to be restricted to southern Africa. In 2008–2009, isolation of EEV in an outbreak reported from Israel demonstrated the emergence of this pathogen into new niches. Indeed, testing in retrospect sera samples revealed that EEV had already been circulating outside of South Africa since 2001. Although EEV normally does not cause severe clinical disease, it should be considered important since it may indicate the possible spread of other related, much more pathogenic viruses, such as African horse sickness virus (AHSV). The spread of EEV from South Africa to central Africa, the Middle East and India is an example of the possible emergence of new pathogens in new niches, as was seen in the case of West Nile virus, and should be a reminder not to limit the differential list when facing a possible outbreak or a cluster of clinical cases. This review summarizes current knowledge regarding EEV structure, pathogenesis, clinical significance, and epidemiology.

## 1. Introduction

Equine encephalosis (EE) is an arthropod-borne, noncontagious, febrile disease of horses. It was first described over a century ago by Theiler, as a mild form of African horse sickness (AHS), under the name “equine ephemeral fever” [1], and was first isolated in 1967 in South Africa from a thoroughbred mare named Cascara that died following febrile nervous disease [2]. The disease is caused by equine encephalosis virus (EEV), an *Orbivirus* of the Reoviridae family, closely related to several other important pathogenic and emerging viruses affecting livestock, including bluetongue virus (BTV), African horse sickness virus (AHSV), and epizootic hemorrhagic disease virus (EHDV), all transmitted by *Culicoides* species [3].

The clinical significance of EEV is probably low, as it usually manifests as mild, transient, febrile disease, which is rarely fatal [3,4]. The risk factors for infection and vector species are similar to those of AHSV, and both viruses usually circulate in the same areas [5,6,7]. Although EEV was considered to be endemic only in southern Africa, reports of its presence in other areas have been accumulating for over a decade [4,8,9,10,11]. These reports coincide with the spread and emergence of other *Orbiviruses* in Asia and Europe due to the combination of animal transport and climate changes leading to changes in *Culicoides* habitat [3,7]. Since EEV is less pathogenic, it may be more easily introduced into new areas and may serve as an indicator of the potential spread of other more clinically important *Orbiviruses,* including AHSV [12].

## 2. Etiology

EEV is an arbovirus of the genus *Orbivirus*, subfamily Sedoreovirinae, and family Reoviridae, transmitted by hematophagous *Culicoides* spp. [13]. The genus *Orbivirus* consists of over 20 serogroups and is the largest genus within the family Reoviridae [13]. Within the EEV serogroup, seven serotypes (EEV-1–7) have been identified to date [14].

**Figure 1:**
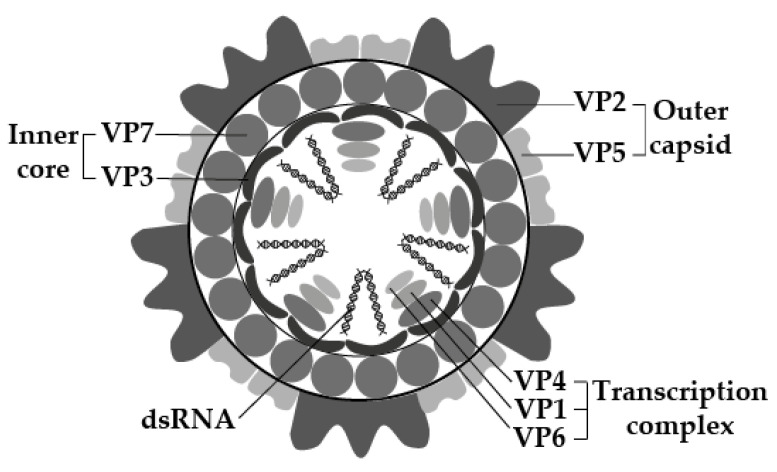
The molecular structure of EEV, according to electron microscopy and molecular studies of EEV, and closely related *Orbiviruses*. Visualization was based on the work of [13,16] and created using Adobe Illustrator 25.4.1© (Adobe Inc., Mountain View, CA, USA).

The viral genome consists of 10 segments of linear double-stranded RNA (dsRNA), surrounded by three layers of capsid proteins, forming a double-layered core particle or inner capsid, surrounded by an outer capsid layer. Virus particles are 60–80 nm in diameter, have icosahedral symmetry, and appear spherical in shape [13,15] (Figure 1).

Similar to other *Orbiviruses*, EEV has seven structural proteins (VP1–7) and four non-structural proteins (NS1–3, NS3a) [13,15,17] (Figure 1, Table 1). The structural proteins include four major capsid proteins (VP2, VP3, VP5, and VP7) and three minor proteins (VP1, VP4, and VP6), with molecular mass (Mr), which ranges between 36,000 and 120,000 [15,18]. The double-layered inner core comprises minor proteins VP1, VP4 and VP6, enwrapped by the major proteins VP3 and VP7. The minor proteins have enzymatic activities involved in viral replication and transcription [13,15,17]. The outer capsid layer comprises two proteins, VP2 and VP5, which are involved in cell attachment and penetration (along with VP7) and possibly in the determination of virulence [13,15,17,18]. Both VP2 and P7 are immunodominant, with VP2 being highly variable and determining EEV serotype [14,15,19].

The 5′- (5′-GUU(U/A)) and 3′- (A(U/A/G)(A/U/C)GUUAC-3′) terminal sequences of gene segments are conserved for all segments within the EEV serogroup [13,15]. Each genomic segment has a single open reading frame (ORF) (Table 1). Seg-9 and Seg-10 mRNAs are translated from either of two in-frame AUG codons (VP6/VP6A, NS3/NS3A); however, the significance of these different translation products is unclear [13] (Table 1). Genomic segments 3, 5, and 9 are serogroup specific and highly conserved between EEV serotypes and, therefore, may be used to distinguish between EEV and other closely related *Orbiviruses* [18,19]. Seg-2, encoding VP2, shows sequence variations that correlate with the virus-serotype [19]. The smallest viral genome segment, Seg-10, encodes NS3/NS3A, which mediates viral release from infected cells and may determine virulence and vector competence. The EEV NS3 gene and protein have a higher level of variation than in other *Orbiviruses*, and phylogenetic studies identified two distinct clusters that correspond with the geographical distribution of different species of *Culicoides* vectors [20]. The ability of *Orbiviruses* to undergo gene reassortment within a single serogroup has resulted in the absence of correlation between virus serotypes and sequence variations in other genomic segments [15,19].

The pathogenesis of EEV infection and replication is similar to other *Orbiviruses*, and involves: (1) cell attachment and penetration, which occurs soon after inoculation (and involve VP2); (2) uncoating and formation of replicative complexes after entry into the cell, the virus is enclosed in endosomes, in which the outer capsid is removed (involving VP5), resulting in the release of transcriptionally active core particles into the cytoplasm; (3) formation of cellular tubules (consisting NS1 and involving VP3 and VP7) with unknown functions that possibly interact with the cellular cytoskeleton, and formation of virus inclusion bodies (containing a different combination of virus particles, with main involvement of NS2); and (4) movement of virus and its release from the cell surface (involving NS3/NS3A [15,21].

Molecular characterization on EEV was based mostly on the variable proteins/genomic sequences VP2 (Seg-2) and NS3 (Seg-10). Seven EEV serotypes have been characterized in South Africa based on the variable protein VP2 (and corresponding genomic sequence Seg-2). The serotypes were assigned numeric values based on the alphabetic order of the location in South Africa where the reference strain originated, namely: EEV-1 (Bryanston, 1976), EEV-2 (Cascara, 1967), EEV-3 (Gamil, 1971), EEV-4 (Kaalplaas, 1974), EEV-5 (Kyalami, 1974), EEV-6 (Potchefstroom, 1991), and EEV-7 (E21/20, 2000) [14]. Phylogenetic analysis of the NS3 gene of South African EEV isolates grouped them into two clusters, which differ by up to 16.7% in amino acid sequence identity. Cluster A included serotypes EEV-1, 2, 4, and 7, and cluster B included serotypes EEV-3, 5, and 6, corresponding to the geographical distribution of the isolates [20].

## 3. Epidemiology

**Figure 2:**
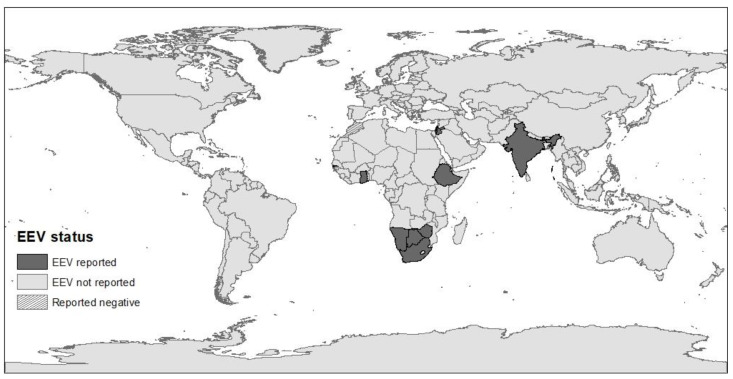
The global distribution of EEV, according to serological and molecular studies. The map was constructed using ArcMap (ArcGIS Desktop 10.6.1, Esri Inc.©, Redlands, CA, USA).

Until 2008, EE had only been reported in southern Africa. Since its first isolation in 1967, additional isolations and epidemiological surveys demonstrated the widespread circulation of EEV in horses and donkeys in South Africa and identified seven serotypes [5,14,22,23]. During the 1990s, EEV seropositivity has also been described in donkeys and zebras in South Africa and neighboring Botswana, Kenya, and Namibia [23,24,25,26]. In 2008, EEV was isolated in Israel during an outbreak of febrile disease in horses [9,27], and further serological studies demonstrated that the virus has been circulating in Israel since 2001 [28] and that it is also present in neighboring Palestinian Authority and Jordan [29]. Following the initial report from Israel demonstrating EEV outside southern Africa, sero-epidemiological studies revealed the virus is endemic in eastern and western African countries, including Ghana (2010), Gambia (2009), Ethiopia (2008), and Zimbabwe (since 1999) [8,10]. However, none of 120 horses sampled in Morocco (during 2008) were found seropositive for EEV, suggesting that the Sahara Desert may serve as a geographical barrier to the spread of the virus [10]. In addition, EEV was isolated in India from a horse that died in 2008 following febrile disease and identified with the help of next-generation sequencing [11]. This clinical case was the only fatal case during an outbreak of febrile disease on the farm, and since that was the first report of EEV in India, its prevalence or spread in the area is yet unknown [11] (Figure 2).

EEV is endemic in Africa and in the Middle East, with seroprevalence ranging between 60% and 100% in Gambia, Ghana, Ethiopia, Israel, South Africa, and Zimbabwe [5,6,8,10,29]. Studies from South Africa and Israel revealed fluctuations in the annual seroprevalence and incidence of EEV, which may be influenced by weather, climate, herd immunity, and the distribution of *Culicoides* vector species [5,6,22,23]. Spatial and temporal studies from South Africa showed that EEV seroprevalence and the abundance of specific EEV serotypes differ between geographical provinces, but also between districts within the same province [5,22,23]. All EEV serotypes have been identified in all South African provinces, but the relative abundance of each serotype varied between areas and even between farms. In each area and season, there was usually one predominant circulating serotype (with demonstrated seroconversion), while others were only isolated sporadically [5,14,22]. However, similar serotypes were identified in horses and *Culicoides* in the same area [5]. It has been demonstrated that individual horses can be simultaneously seropositive to several serotypes, which indicates that there is no sufficient immunological cross-protection between serotypes against infection [5,14,22,30].

Sequence analyses of genomic segments provide additional information to serotype classification (which is based on serologic reaction to VP2 protein). Sequencing of the VP2 serotype 4 gene of isolates from Gambia and Israel found them unique to South African isolates and grouped them together, suggesting a common source outside of South Africa [10,27]. Characterization of NS3 genomic sequences of serotype 1 from an outbreak in Western Cape (1999) encoded identical proteins, which indicates a high level of conservation during the outbreak [20]. Although NS3 did not cluster according to geographic location [20,31], close phylogenetic relationships were found between EEV isolates from horses and from *Culicoides* during the same period and area [32], supporting the correlation found between the geographical distribution of EEV serotypes in horses and *Culicoides* and NS3 phylogenetic clusters [5]. Recent analysis on the full sequences of all 10 genome segments of 17 EEV isolates of all serotypes revealed widespread reassortment in EEV strains, with unique segments that may be associated with geographic location [32]. For example, the EEV isolate from India was classified as serotype 1 according to its VP2 sequence but was similar to serotype 6 according to its NS3 and VP1 sequences and had a unique combination of other segments than any of the South African isolates [11,32]. Field isolates from clinical cases had as little as 81.6% amino acid similarity to their corresponding serotype reference strains. This limited similarity may suggest genetic drift, which may possibly lead to immune evasion [32].

EEV is biologically transmitted by *Culicoides* biting midges, of which *C. imicola* and *C. bolitinos* have been shown to play an important role in South Africa [5,33,34]. The *Culicoides* genus includes over 1400 described species that inhabit a wide range of habitats. Only a small proportion of these species are known vectors of *Orbiviruses*; however, most studies only focus on certain species [35,36]. In South Africa, EEV was identified in *Culicoides* blood pools of several species [5,31,33,34,37], with high recovery rates (VRR) immediately following feeding (81.9%) and virus survival and multiplication demonstrated in five of 19 species tested following incubation for 10 days [5]. The mean levels of viral replication differed significantly between EEV serotypes and *Culicoides* species, suggesting that certain species have served as better vectors to specific serotypes [5] (Table 2).

In South Africa, *C. imicola* and *C. bolitinos* are the most abundant and widespread species and were identified as important vectors of EEV and other *Orbiviruses* such as BTV and AHSV. The higher vector competence of these two species for EEV-1 correlates with the high field recovery rate of this serotype from horses in South Africa [5,14,22]. The differences in prevalence and rate of exposure to individual serotypes within and between regions in South Africa may be attributed to the differences in their spatial and temporal distribution of certain *Culicoides* species, in combination with the differences in the competence of these vectors to specific serotypes. *C. imicola* is the main species in the northern regions of South Africa, which correspond with NS3 gene cluster B, while *C. bolitinos* is more abundant in the southern districts, corresponding with NS3 gene cluster A [5,20,38]. In addition, the low EEV seroprevalence in the Western Cape Province in South Africa could be attributed to the lower abundance of *C. imicola* in this region [5].

The epidemiology of *Culicoides*-borne diseases is often complex and involves multiple vectors and hosts within a geographical region [35]. *Culicoides* species have a worldwide distribution (except for Antarctica and New Zealand). The success of *Culicoides* to serve as vectors is related to their population size and means of dispersal, which are highly influenced by climate and weather [39]. Therefore, climatic differences, which affect the spatial and temporal distribution of *Culicoides*, may influence the prevalence of certain EEV serotypes in specific geographical areas or during an outbreak [5]. Although EEV is endemic in South Africa and normally has minimal clinical significance, local outbreaks are sometimes reported [14,30]. Long-term epidemiological studies from South Africa, Israel, and Zimbabwe demonstrated fluctuation in the rate of infection between years [6,8,22]. In South Africa, the annual seroprevalence in yearling foals ranged between 3.6% and 34.7% [22] and varied between EEV serotypes [14,22]. These fluctuations may reflect changes in vector distribution. Seasonal drought followed by heavy rainfall had been shown to increase the chance of arboviral diseases, including the EE outbreak in Israel in 2008 [6] and AHS outbreaks in South Africa [22,33,40]. This association was mainly explained by an effect of water deficit on the environment, altering the relationships between vectors and hosts (as water sources may be more available in farms), but might also be the result of changes in the vectorial capacity of the insects, inferred by drought [41]. Since several important *Orbiviruses* share the same *Culicoides* vectors, their spread and outbreaks often coincide. Several studies demonstrated similar infection patterns of EEV and AHSV in equids, with usually higher prevalence of EEV than of AHSV in both hosts and vectors [8,23,24,25,37], and higher incidence of EEV clinical cases was detected during outbreaks of AHS or other arboviruses in certain districts of South Africa [32]. Therefore, EEV surveillance may be important to infer on the circulation of other *Orbiviruses*, especially AHSV, against which many horses in southern Africa area are routinely vaccinated. The global changes in climate have led to changes in vectors’ habitat and range and to the expansion in the habitat of *Culicoides* species, namely *C. imicola*, which is a major vector of EEV, AHSV, BTV, and other veterinary important *Orbiviruses*. These changes led to the emergence and spread of various *Orbiviruses* into more temperate regions and to an increase in global incidence and virus diversity [35,36,42]. Several studies aimed to evaluate the risk for the introduction of EEV into European countries pointed at two possible routes of introduction: importation of infected animals or importation of infected vectors, with the former being more probable [12,43,44]. In addition, the risk of the introduction of EEV through an infected host was higher than that of AHSV [12,43].

Herd immunity has also been suggested to serve as a protective factor against EEV infection. In Israel, lower annual incidence was recorded in farms with initial higher seroprevalence [6]. In South Africa, a possible pattern was suggested for predominant serotypes within an area in which high prevalence was recorded for a season or two, followed by a dramatic reduction in the incidence in the following year [22]. Immunity seems to be serotype specific, and maternal immunity (which is estimated to last until the age of 5–6 months) does not seem to prevent EEV infection of foals, both because of the variable composition of serotype-specific maternal antibodies and diminished maternal antibody levels prior to the high-risk season (the end of the rainy season) [14,22,30]. Transplacental transmission is probably not a major route of transmission, although abortions have been reported as a consequence of EEV infection, and the virus has been isolated from a placenta of a mare with a fatal case of EEV. Vertical transmission in the vertebrate host has been reported for BTV but not for AHSV [32].

EEV has been reported to infect various equid species, including horses, donkeys, and zebras in southern Africa, with similar prevalence rates and serotype distribution [8,23,24,25]. Donkeys are considered to be more resistant to clinical disease, are widely dispersed over various ecological zones, and are usually more susceptible to the presence of insect vectors. Therefore, donkeys are considered to be ideal sentinels for both EEV and AHSV [14,23]. A high-resolution study of zebras at Kruger National Park (KNP) demonstrated continued exposure to EEV throughout the year, attributed to the unbroken presence of the vector throughout the year in the subtropical climate. It has been suggested that zebras may play a role in the persistence and over-wintering of EEV in the area when *Culicoides* abundance is low due to the combination of high numbers of susceptible foals and sufficient numbers of *Culicoides* vectors during winter [24]. EEV had also been serologically detected in four elephants. This observation might be incidental or false (due to the non-specific reaction of elephant sera in serological test), and their role in the circulation of EEV in the area is yet undetermined [25].

## 4. Clinical Disease

The clinical significance of EEV infection is difficult to determine but is probably low. Generally, EEV is associated with mild or subclinical disease in horses with low mortality rates [3,4]. Characteristic clinical presentation of symptomatic horses consists of a short period (typically two to five days) of fluctuating fever and inappetence, sometimes accompanied by tachycardia and tachypnea [27]. Currently, there is no evidence of the zoonotic potential of EEV.

The name “equine encephalosis” is misleading, as the disease is not primarily neurologic. The name was given when the virus was first isolated from the organs of a mare that died during an outbreak at the farm in which three mares were affected, and two died. All three mares suffered from a peracute, febrile, nervous disease, and both fatal cases were diagnosed with edema and congestion of the brain, focal catarral enteritis, and mild fatty generation of the liver [2,14]. In the following years, EEV was isolated from horses exhibiting a variety of clinical signs including, fever, inappetence, central nervous system signs including severe ataxia, stiffness, changes in temperament and convulsions, respiratory signs including nasal discharge, enteritis, cardiac failure, liver damage and icterus, abortion (at 5–6 months), conjunctivitis, and swelling of the neck, lips or eyelids [4,9,11,14,28]. However, only limited numbers of clinical cases have been described, despite the high seroprevalence of EEV in South Africa suggesting that most cases are subclinical [30].

The clinical signs of EE are non-specific and could be easily confused with that of other viruses. Initially, EE symptoms were described as a mild manifestation of AHS, and EE outbreaks often coincide with outbreaks of AHS or other arboviruses. Therefore, to confirm the diagnosis of EEV as the cause of disease, the virus should be directly identified, and other potential pathogens should be ruled out [28,32]. In a recent analysis of 1523 samples from horses in South Africa presenting neurological, febrile, respiratory signs, or sudden death, 7.3% (111 horses) were infected with EEV (as diagnosed by real-time reverse-transcriptase PCR, rRT-PCR). Of these EEV-positive horses, 17 were co-infected with other arboviruses (AHSV, West Nile virus, or Middelburg virus). Clinical signs that were significantly associated with EEV-positive cases were fever, dyspnea, and icterus. In contrast, neurological signs (and specifically ataxia) and case fatality (including euthanasia) were inversely associated with EEV infection. Although 47.7% of EEV-positive horses had neurological abnormalities (some of which were co-infected with other viruses), only 9% had fatal outcomes [32]. In general, fatality rates following EEV infection are relatively low and estimated at 0% to 5% of clinical cases [4,9].

There is no sufficient data of possible associations between specific clinical signs and EEV serotypes. The “original” neurologic syndrome could only have been experimentally reproduced once, using the EEV-2 (Cascara) serotype [14]. Full genome sequences obtained from six clinical cases (three neurologic, one febrile, one dyspneic, and one abortion) classified five as EEV-1, while the horse with respiratory signs was infected with EEV-4 [32]. Since EEV-1 is the most prevalent serotype in South Africa [14,22], it is difficult to infer from these findings differences in pathogenicity between genotypes. In general, the negative association between neurological signs and case fatality and EEV in clinical cases [32] suggests that EEV is probably not a major cause of neurological disease or case fatality in endemic areas.

## 5. Treatment, Prevention, and Control

No specific treatment is available against EEV. Most symptomatic cases recover with no complications. Supportive treatment may be administered to decrease fever and inflammation or relief other clinical signs. No vaccine is currently available against EEV [4,27].

EEV is considered noncontagious, and prevention strategies mainly focus on reducing exposure to *Culicoides* vectors. Vector control is usually based on a combination of mechanical, chemical, biological, and genetic methods used to limit the vector’s habitat and reduce vector-host contact. Such methods are most relevant to stabled horses and include stabling horses at dusk and dawn (when the vectors are more active), reducing light at night, screening windows, treating or removing animal waste, and using vector repellents on horses and the environment [4,45].

To prevent the introduction of EEV into new areas, transportation restrictions should be applied [45]. However, since EEV has limited veterinary and economic impact, such restrictions are not required to date. Modeling of the possibility of introduction of EEV from endemic countries into Europe demonstrated that control measures prior to exportation, including mostly quarantine and vector control, but also clinical inspection and serological screening, are efficient in reducing the probability of EEV introduction [12,43].

## 6. Diagnosis

Rapid and accurate diagnosis is important, especially during disease outbreaks, with sufficient specificity to distinguish between closely related pathogens in order to implement appropriate treatment and control. Different methods have been developed for the detection and classification of EEV, including virus isolation, serological assays, and molecular assays.

Historically, EEV was identified by virus isolation in baby hamster kidney (BHK) cells, suckling mice brain, embryonated chicken eggs, and Vero cells (African green monkey kidney cells) [2,4,11,19,29,32]. Most experimentally infected cell lines displayed post-infection cytopathic effects [11] and can be used for virus neutralization tests (VNT). However, virus isolation methods are labor intensive and are not very sensitive.

Several serological methods detecting anti-EEV antibodies have been developed, which are most useful for screening and epidemiological surveys. Some of these assays are group-specific enzyme-linked immunosorbent assays (ELISA) detecting antibodies against all EEV serotypes, but not of other *Orbiviruses.* These methods include competitive ELISA (cELISA) and indirect sandwich ELISA (S-ELISA), both having 100% sensitivity and specificity [46,47]. Other methods are serotype specific and used to determine EEV serotype, mainly VNT [14].

Molecular assays are usually very specific and sensitive and are increasingly being used for the identification and classification of EEV, as well as other *Orbiviruses*, at a serogroup and serotype level. Genomic probes have been developed for the detection of EEV. The NS1 (Seg-5) gene was the most sensitive for the detection of EEV at a serogroup level, while the VP2 (Seg-2) gene was serotype specific [18,48]. Real-time reverse-transcriptase polymerase chain reaction (rRT-PCR), using TaqMan probes have been developed to detect EEV at the serogroup level, using VP7 (seg-7) and VP6 (Seg-9) genes, and at the serotype level, using VP2 (seg-2) gene, with high sensitivity, specificity, and efficacy [19,38]. Full and partial genome sequencing (of one or more segments) of EEV have also been used for phylogenetic studies comparing viral species, isolates, or genotypes and are usually used for epidemiological investigations rather than routine clinical detection [11,20,27,32,49]. In some cases, next-generation sequencing was used to identify EEV from isolates or total RNA from clinical cases in new geographical areas, where EEV had not been suspected [9,11].

## 7. The Israeli Perspective

Between October 2008 and January 2009, a febrile horse disease was observed in hundreds of horses in more than 60 equine premises across Israel. Initial serological results indicated that the disease was equine viral arteritis (EVA), but this virus was not isolated, and PCR tests were all negative. Using a novel DNA array technique, with subsequent RT-PCR and sequence analysis in the Veterinary Laboratories Agency (VLA) in the United Kingdom (U.K.), the virus was identified as EEV [9]. This was the first time that this virus was isolated anywhere else north to South Africa. A year later, samples were collected from eight febrile horses in Israel, and cultures from three of these horses were positive for EEV [27]. Phylogenetic analysis of VP2 (Seg-2) showed 92% sequence identity to EEV-3, and the phylogenetic analysis of EEV NS3 (Seg-10) grouped these isolates with other EEV isolates but as a distinct group [27]. Based on these differences, it was speculated that this virus has evolved in the region for a sufficient time to accumulate these changes and was not recently introduced to Israel from South Africa [27]. Indeed, soon afterward, retrospective analysis of sera samples collected from horses in Israel for other reasons revealed anti-EEV antibodies in four of five sera samples that were collected in 2001 [28], and similar isolates have also been characterized in Gambia.

This sequence of events resembles the events leading to the first description of EEV in South Africa in 1967, in which the EEV outbreak was initially described as a mild case of AHS [2] (and later was connected to the first description from 1910 [1]), and its first isolation in India, following an outbreak of febrile disease that led to an investigation during which numerous other pathogens have been ruled out, and only next-generation sequencing of total RNA finally identified EEV as the cause of disease [11]. The course of these epidemiological investigations demonstrates the diagnostic challenge when encountering a newly introduced or emerging pathogen in a new area, especially when it does not have characteristic clinical signs.

During the 2008 outbreak in Israel in which more than 60 stables were affected, clinical signs included mainly: raised body temperature, pulse, and respiratory rates, unrest, and decreased appetite. Although morbidity was high (and reached 100% in some of the farms) and was reported in horses from different breeds, ages, and sexes, no fatalities were reported [9]. Since this outbreak, EEV has been sporadically diagnosed as a cause of febrile disease, with few local outbreaks. In these diagnosed or related cases, the clinical signs included fever and inappetence for 2–5 days, with complications of respiratory signs or colic in the minority of cases (unpublished data). This observation is contrary to other reports of severe neurological or life-threatening signs during EEV outbreaks [14,32]. It has been proposed that EEV serotypes may differ in their pathogenicity [14,32]; however, no evidence of such differences is currently available. In this respect, it is important to mention that during extensive outbreaks, it is probable that not all clinical cases are the result of the same pathogen, and unless definitively diagnosed, caution should be used when making such interpretations. Based on our experience, in an endemic area, where clinical cases are suspected every year, it seems that the clinical significance of EEV is low, and it is mainly important as a model for the spread of new pathogens to new niches where the vectors are present.

## 8. Conclusions

Although EEV is probably not of major clinical importance, its emergence in areas outside southern Africa may precede the spread of other, closely related, *Culicoides*-borne pathogens, such as AHSV and BTV. For the time being, the virus has been reported from southern and central Africa, Israel, and India but has not spread to moderate climate countries in Europe or the Americas. The lower virulence of this virus, in combination with uncharacteristic clinical signs, makes its diagnosis challenging, especially in areas when it is not known to be endemic. Future research is needed to better understand the epidemiology and pathogenesis of EEV, as well as the dynamics of the circulation of various *Culicoides*-borne arboviruses.

## Figures and Tables

**Table 1:** Genome segments and protein encoded by each segment of EEV (similar to other *Orbiviruses*).

Genome Segment	Protein	Function
Seg-1	VP1	RNA-dependent RNA polymerase
Seg-2	VP2	Protein of the outer layer of the outer capsid, involves in cell attachment, most variable, determines serotype
Seg-3	VP3	Innermost protein capsid shell
Seg-4	VP4	Capping enzyme
Seg-5	NS1	Forms tubules of unknown function
Seg-6	VP5	Inner layer of outer capsid, involves in cell penetration
Seg-7	VP7	Protein of the outer core surface, involves in cell entry, immunodominant
Seg-8	NS2	Inclusion body matrix protein
Seg-9	VP6/VP6A	Helicase
Seg-10	NS3/NS3A	Membrane protein, involves in cell exit, variable

**Table 2:** Survival of EEV serotypes in various *Culicoides* species following 10 days incubation at 23.5 °C after membrane feeding of infected blood [5].

*Culicoides* spp.	EEV Serotype Survival
*C. imicola*	EEV-1 >> 4 > 2,5 > 3 > 6
*C. bolitinos*	EEV-2 > 1 >> 4 > 6
*C. leucostictus*	EEV-1 > 2
*C. magnus*	EEV-1
*C. zuluensis*	EEV-2

## Data Availability

Not applicable.
